# Deconvolution of the Cellular Force-Generating Subsystems that Govern Cytokinesis Furrow Ingression

**DOI:** 10.1371/journal.pcbi.1002467

**Published:** 2012-04-26

**Authors:** Christopher C. Poirier, Win Pin Ng, Douglas N. Robinson, Pablo A. Iglesias

**Affiliations:** 1 Department of Electrical and Computer Engineering, Johns Hopkins University, Baltimore, Maryland, United States of America; 2 Department of Cell Biology, Johns Hopkins University School of Medicine, Baltimore, Maryland, United States of America; 3 Department of Biomedical Engineering, Johns Hopkins University School of Medicine, Baltimore, Maryland, United States of America; 4 Department of Chemical and Biomolecular Engineering, Johns Hopkins University, Baltimore, Maryland, United States of America; 5 Department of Pharmacology and Molecular Sciences, Johns Hopkins University School of Medicine, Baltimore, Maryland, United States of America; North Carolina State University, United States of America

## Abstract

Cytokinesis occurs through the coordinated action of several biochemically-mediated stresses acting on the cytoskeleton. Here, we develop a computational model of cellular mechanics, and using a large number of experimentally measured biophysical parameters, we simulate cell division under a number of different scenarios. We demonstrate that traction-mediated protrusive forces or contractile forces due to myosin II are sufficient to initiate furrow ingression. Furthermore, we show that passive forces due to the cell's cortical tension and surface curvature allow the furrow to complete ingression. We compare quantitatively the furrow thinning trajectories obtained from simulation with those observed experimentally in both wild-type and *myosin II* null *Dictyostelium* cells. Our simulations highlight the relative contributions of different biomechanical subsystems to cell shape progression during cell division.

## Author Summary

Cytokinesis, the physical separation of a mother cell into two daughter cells, requires force to deform the cell. Though there is ample evidence in many systems that myosin II provides some of this force, it is also well known that some cell types can divide in the absence of myosin II. To elucidate the mechanisms by which cells control furrow ingression, we developed a computational model of cellular dynamics during cytokinesis in the social amoeba, *Dictyostelium discoideum*. We took advantage of a large number of experimentally measured parameters and well-characterized furrow ingression dynamics for a number of different strains. Our simulations demonstrate that there are distinct phases of cytokinesis. Myosin II plays a role providing the stress that initiates furrow ingression. In its absence, however, this force can be supplied by a combination of adhesion and protrusion-mediated stresses. Thereafter, Laplace-like pressures take over and provide stresses that enable the cell to divide. Overall, we show how various mechanical parameters quantitatively impact furrow ingression kinetics, accounting for the cytokinesis dynamics of wild type and mutant cell-lines.

## Introduction

Cytokinesis, the separation of a mother cell into two daughter cells, is a highly stereotypical cell shape change. During most mitotic events, cytokinesis requires the careful orchestration of many cellular systems to ensure that the cell separates the genomic material into two genetically equivalent daughter cells [1], [2]. However, the core process can be altered to produce asymmetric cell division events in which the daughter cells differ dramatically in size and/or cell differentiation fate [3], [4], [5].

For cytokinesis, myosin II is a key but non-essential mechanoenzyme that converts the energy of ATP hydrolysis into mechanical work [6]. Myosin II works on the actin network to alter the cell's mechanical properties in complex ways. By pulling on the filaments, myosin II can slide the polymers. This activity is the core of the traditional contractile ring model in which myosin II slides filaments, contracting the ring in a manner analogous to the contracting muscle sarcomere [7]. However, the actin polymers are held together by various actin crosslinking proteins, each with its own unique kinetic characteristics, force-sensitivity, and concentration. Thus, myosin II pulls on anchored actin filaments, leading to an effective tension due to the stalling of the myosin II motor in the isometric state [8], [9]. As a result, myosin II is not rate-limiting for furrow ingression, and previous analyses have indicated that the furrow ingresses some 30–50-fold more slowly than predicted from the myosin II unloaded actin filament sliding velocity [10].

Ultimately, appreciating how the cell integrates three properties – biochemistry, mechanics and morphology – is the crux of understanding all cell shape changes. Because cytokinesis proceeds through genetic strain-specific geometries and characteristic dynamics, it is particularly well suited for studying how cell shape changes arise from biochemical mechanisms. This view has led to the concept that cytokinesis requires the function of the entire cortex and cytoplasm and is governed by two basic modules, global and equatorial actin-associated proteins [9]. Myosin II is found throughout the cortex but in a roughly two-fold concentration gradient between the equatorial and polar cortical domains [11]. The myosin II-mediated force generation is only one of several major mechanical systems of the cell. Two other systems include polar protrusive forces and the viscoelasticity of the cytoskeleton [8], [12]. Another major mechanical component is derived from the cell's surface cortical tension and surface curvature, which leads to fluid pressure differentials that make cytokinesis in particular, and cell shape change in general, hydrodynamic in character. These pressure differentials lead to net flows of cytoplasm away from regions of high surface curvature to regions of lower curvature, allowing the furrow to ingress with dynamics that are controlled by the fluid dynamical and mechanical features of the cell [10]. Here, we present a computational model that demonstrates how the cell's major mechanical subsystems are integrated to drive and control cytokinesis. In particular, the model considers these separate mechanical subsystems, and explains the dynamical features of wild type and mutant cytokinesis events. Most significantly, the model demonstrates that these biomechanical systems are sufficient to explain cytokinesis.

## Results

**Figure 1:**
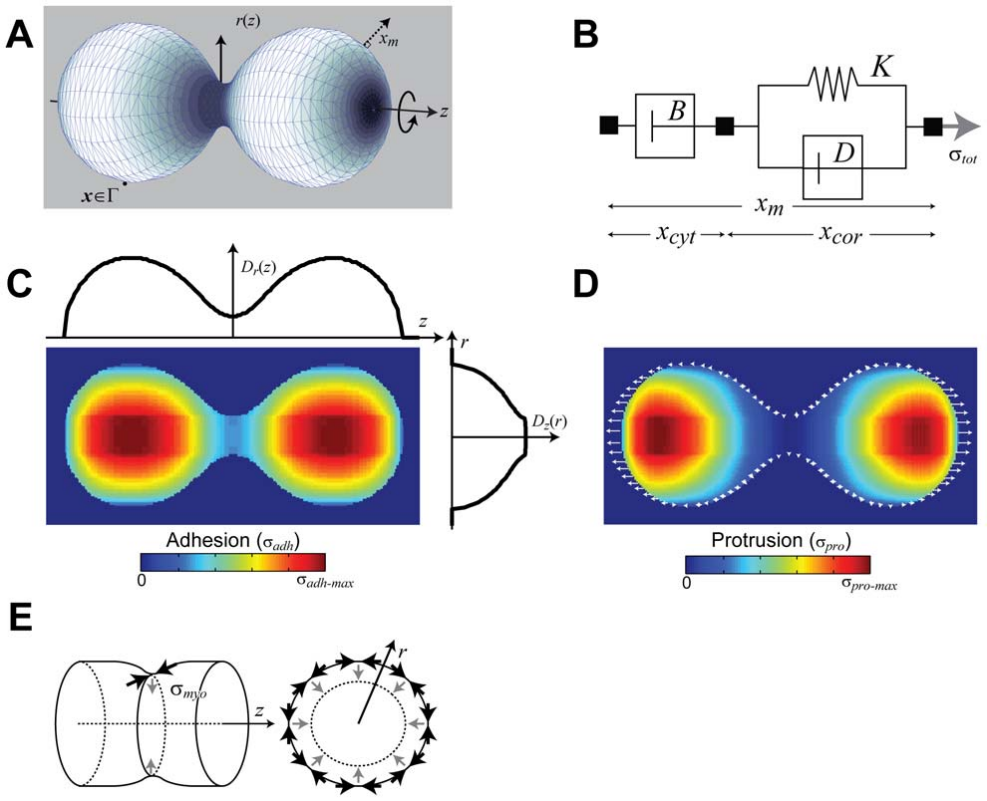
Level set model geometry and stress distribution. A. The cell model assumes cylindrical symmetry. Points on the cell boundary (***x***∈Γ) are obtained implicitly. B. Using a viscoelastic description of the cell (Equation 3), cell boundary/membrane displacements (*x_m_*) are generated by moving the potential function (φ, not shown) according to the total stress applied, σ*_tot_* (Equation 4). The spring-dashpot (*K*, *D*) elements represent the mostly elastic cortex, which moves a distance *x_cor_*. The viscous component (*B*) represents the cytosol, which moves a distance *x_cyt_*. Values for *K*, *B* and *D* were previously obtained using micropipette aspiration experiments and are given in Table 2. C. Area density maps (*D_r_*(*z*) and *D_z_*(*r*)), obtained by summing the cell area (in the *z-r* plane) one axis at a time (Equation 5). The resultant adhesion map, shown overlaid on the cell shape, is obtained by multiplying these two together. D. Protrusive stress is assume to work in the *z*-direction away from the furrow according to Equation 7, but only the component normal to the boundary is used. E. Geometry of contractile stress. Though myosin II acts radially, its effect is to reduce the circumference, and hence radius. This can be recreated by applying a stress (σ*_myo_*) inwards radially (shown in gray).

**Figure 2:**
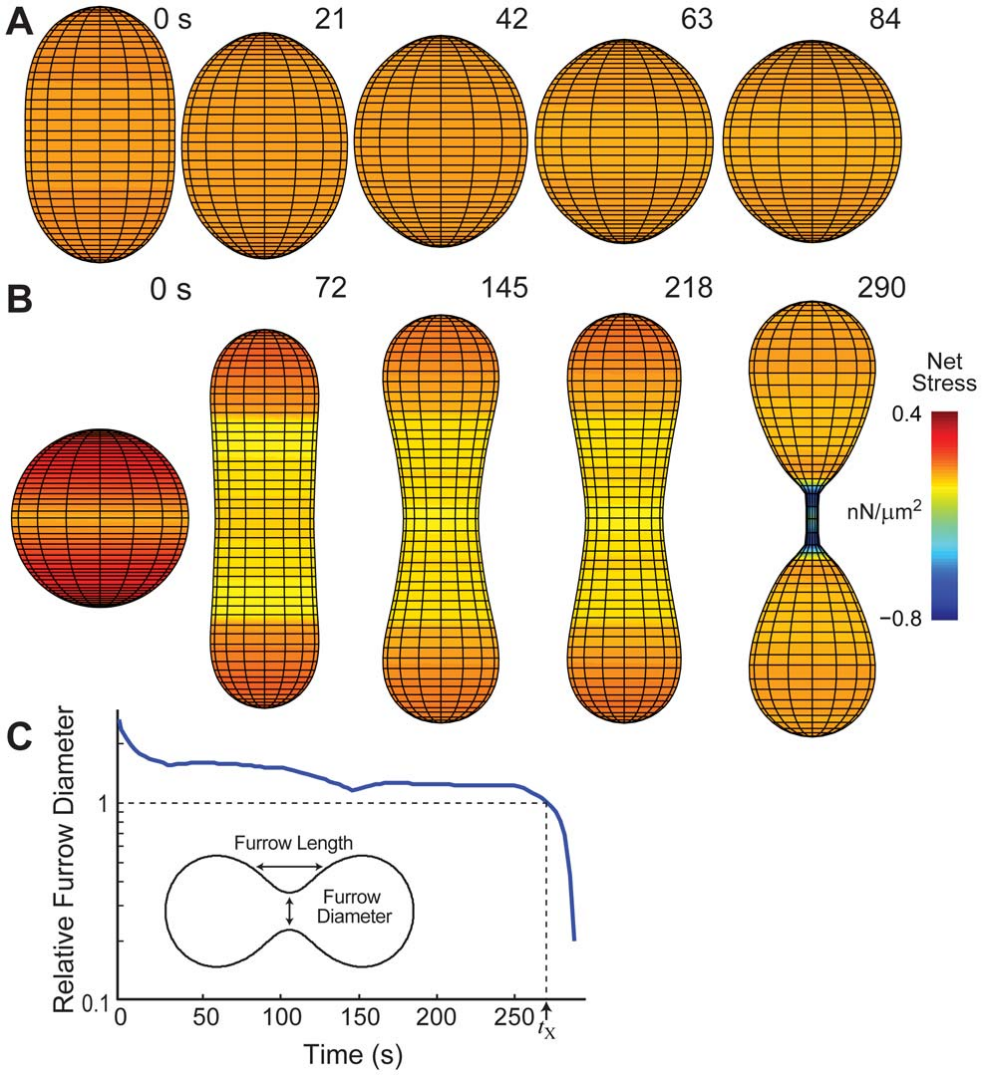
Simulations of interphase cells under various stresses. A. Simulation of a non-adherent cell, initialized as an ellipsoid, experiencing only passive forces. As expected, the cell rounds up relatively quickly. B. Stresses due to adhesion and protrusion were incorporated into the model to simulate traction-mediated cytofission (Video S1). The stress color scale applies for both panels A and B. Negative stress is inward-directed. C. Furrow ingression dynamics of the cell for the simulation in panel B. The point in time when the furrow diameter and length are equal is defined as the cross-over time (*t*_X_) and this distance is known as the cross-over distance. The relative furrow diameter is the ratio of furrow diameter divided by the cross-over distance.

To examine the roles that different subsystems have on shape changes during cytokinesis (Table 1), we developed a viscoelastic mechanical model of the cell into the level set formalism (Methods; Fig. 1). Level sets are a particularly attractive method for simulating large cellular deformations as they represent dynamic surfaces implicitly [13]. Our viscoelastic model (Fig. 1B) was obtained from and verified through experiments using micropipette aspiration [14]. We first considered the simplest case, a non-mitotic cell in a non-adherent environment that experiences only passive forces due to Laplace-like pressure acting normal to the surface. This pressure is proportional to the effective cortical tension and the mean curvature at the surface. Simulations in which we initialized the cell in a non-spherical shape show the cell experiencing greatest force at the regions of high curvature causing a relaxation towards a spherical morphology as might be expected from lack of symmetry breaking active forces (Fig. 2A).

**Table 1:** Simulations considered.

Condition modeled	Stresses included					Mechanics		Simul.
	σ*_adh_*	σ*_pro_*	σ*_myo_*	σ*_ten_*	σ*_vol_*	Δ*ten*	Str. Stiff.	Results
Traction-mediated cytofission	√	√	-	√	√	-	-	Fig. 2B
*myosin II*-null; non-adherent	-	-	-	√	√	√	-	Fig. 3A
*myosin II*-null; adherent	√	√	-	√	√	√	-	Fig. 3B
WT; non-adherent	-	-	√	√	√	√	-	Fig. 4A
WT; adherent	√	√	√	√	√	√	-	Fig. 4B
WT; adherent; strain-stiffening	√	√	√	√	√	√	√	Fig. 4C

### Cells undergo traction-mediated cytofission

We next sought to determine whether our model cells could undergo traction-mediated cytofission, a process whereby multinucleated cells can divide during interphase [15]. We incorporated adhesion into the model taking advantage of recent measurements of the traction experienced by motile *Dictyostelium* cells (Fig. 1C) [16]. Starting from a spherical cell, we applied protrusive forces in directions 180° apart (Fig. 1D). Though this assumption represents a geometrical simplification that allows us to take advantage of cylindrical symmetry, the amount of force is proportional to the cross-sectional area of the cell (initially a circle) and is representative of the protrusive force experienced by a cell that makes a hemispherical contact with the substrate. This force led to relatively slow cell elongation and initially, concomitant slow furrow ingression (Fig. 2B; Video S1). However, as the furrow narrowed, the cortical tension combined with an increase in local curvature to amplify the local stress. This, in turn, accelerated the rate of furrow ingression, increasing the local curvature further. This positive feedback loop caused a drastic pinching of the furrow, leading to daughter cell separation (Fig. 2B,C). It must be noted that the mean curvature depends on the 3-D nature of the local geometry which involves both axial and radial components. The former is decreasing as the furrow ingresses, but the latter increases greatly during constriction.

Experimentally, it is documented that separate molecular mechanisms are needed to promote the scission of the bridge joining the two daughter cells [17], [18]. Furthermore, measurements of the furrow ingression dynamics show the existence of a bridge-dwelling step that is quantitatively separable from the mechanical stresses that drive furrow ingression [10]. For these reasons, we did not attempt to simulate the final bridge severing and stopped the simulations at this point.

### Spatial heterogeneities in cortical tension can initiate cell division, but only in adherent cells

**Figure 3:**
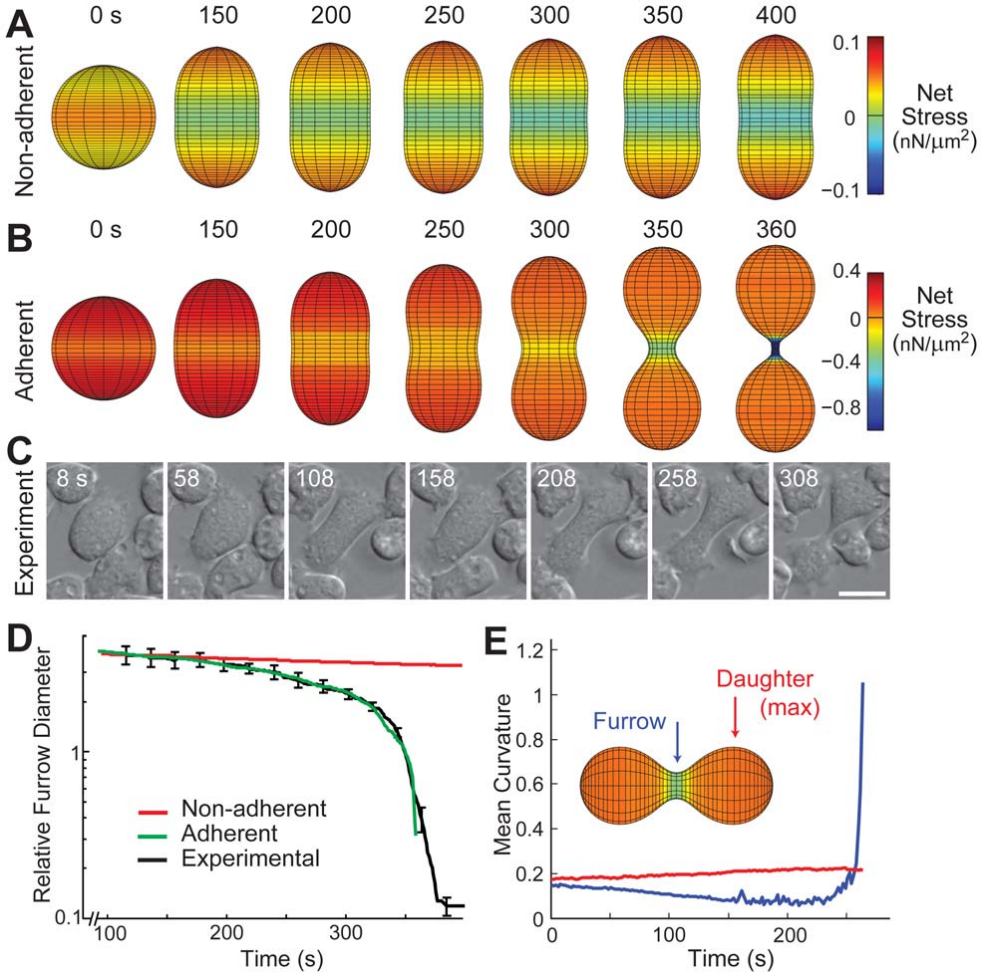
Simulation of *myoII* null cells. Morphological changes in a model where there is a spatial difference in cortical tension for both non-adherent (A) and adherent (B) cells (Video S2). Simulation times are from the initial spherical shape. The distribution of the stresses in the adherent case is shown in Fig. S5. C. Experimental data are taken from *myoII* null cells dividing on a surface. Experimental times are from Video S3. Scale bar denotes 10 µm. D. Comparison of the furrow thinning trajectory. The experimental data represents mean ± SEM and are taken from reference [12]. To compare the shapes at comparable times, time is rescaled so that the cross-over points coincided (Methods). E. Curvature in a simulation of adherent cells. The curvature at the furrow initially decreases slowly but reaches a minimum before increasing. This causes the stress to increase further increasing curvature and thereby closing a positive feedback loop which leads to rapid cell ingression. The curvature of the daughter cell changes relatively little.

The rapid rate at which curvature-induced differences in cortical tension enabled furrow ingression in the previous simulation led us to posit whether spatial differences in the material properties of the cell could initiate ingression and eventually give rise to sufficient forces leading to cell division. Using micropipette aspiration, we previously measured the effective cortical tension under several contrasting conditions, including interphase vs. mitotic, WT vs. *myoII* null, and furrow vs. polar regions and demonstrated that the furrow exhibits a 20–30% higher effective cortical tension relative to the poles [8], [12]. We incorporated this heterogeneity into the model and simulated cytokinesis in non-adherent (Fig. 3A) and adherent conditions (Fig. 3B; Fig. S5; Video S2). In both cases, heterogeneity in effective cortical tension and the resultant difference in Laplace-like pressures cause furrow ingression. In non-adherent cells, however, furrow ingression stops shortly after commencing and is not sufficient to cause further ingression or cell division. By increasing the difference in effective cortical tension, we were able to achieve cell division, but this required non-physiological differences (3–10 fold) in effective cortical tension between pole and equator (not shown). On the other hand, the addition of transient adhesive and protrusive forces led to successful cell division (Fig. 3B). These forces appear to be required to induce a sufficient change in morphology (specifically, curvature) from which cortical tension can complete furrow ingression.

It is well documented that *Dictyostelium* cells lacking functional myosin II cannot divide in suspension, but successfully divide when placed on an adhesive surface [19]; similar observations have been made of mammalian cell culture cells [20] (Fig. 3C). Though this division is similar to those observed in WT cells, there are some significant differences. The furrow ingression dynamics (quantified as the time-dependent change in the relative furrow diameter) display biphasic behavior, in which a slow phase of ingression is followed by a rapid one [10]. We found strong agreement between the furrow-thinning dynamics predicted by our simulation and those measured experimentally in *myoII* null cells (Fig. 3D; Video S3). Plotting the curvature at furrow and poles during division, it is clear that the second rapid phase of furrow ingression can be attributed to the large increase in force that comes from an increase in mean curvature at the furrow (Fig. 3E) as the radial component of curvature begins to dominate. There are some noticeable differences in the shapes of the simulated cells when compared to the *myoII* null cells (Fig. 3C,D). In real cells, protrusions are more “stochastic” causing ruffling at the poles. In our model, protrusive stresses are applied uniformly across the boundary and lead to a rounded shape. The treatment of adhesions is also likely to cause some of these differences. In our model, adhesion is modeled as a homogeneous friction, whereas in cells it is more likely to be localized, and this will affect the shape [21]. Furthermore, in *myoII* null cells, cortexillin I is not as focused in the cleavage furrow as in wild-type cells [22], [23], which could broaden the zone of increased elasticity

### Contractile force from myosin II can also drive furrow ingression

**Figure 4:**
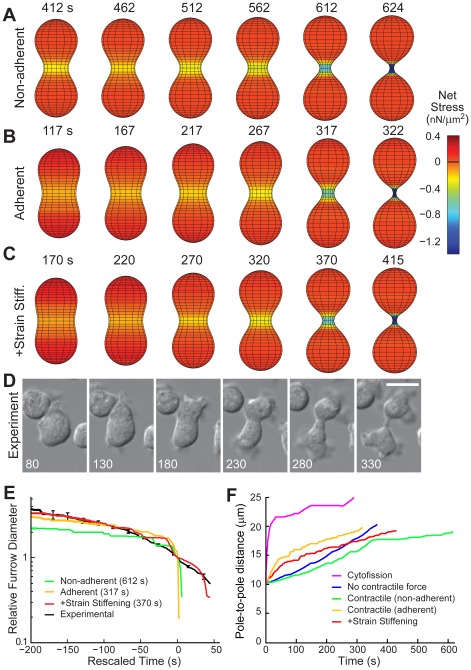
Cell division in the presence of a contractile force. Simulation of dividing cells in both non-adherent (A; Video S4) and adherent conditions (B; Video S5). In the latter we also considered the effect of strain-stiffening as defined by Equation 11 (C; Video S6). Simulation times are from the initial spherical shape. D. Experimental comparison is with WT cells. Experimental times are from Video S7. Scale bar denotes 10 µm. E. Comparison of furrow thinning trajectory. Experimental data represent the mean ± SEM and are taken from reference [12]. We rescaled the time axis to compare the shapes at comparable times, by shifting the time so that the cross-over times are denoted as 0 s (Methods). The elapsed time between the start of the simulation and the cross-over time for each simulation is given in the legend. F. Pole-to-pole distance as a function of time.

Having established that material heterogeneities cannot initiate division but can provide the required force to finish it, we next considered the effect of a myosin II contractile force in our simulations. To this end, we determined the location of myosin II motors from fluorescent images of GFP-myosin II (Fig. S1) and distributed a contractile force temporally and spatially based on the measured distribution of myosin II motors in the cortex (Methods). Incorporating this contractile force in simulations of non-adherent cells led to successful division (Fig. 4A; Video S4). This demonstrates that a cell in suspension can initiate division by substituting the initial ingression provided by adhesion and protrusion on surfaces by myosin II constriction at the furrow. We also observed division in simulations of adherent cells (Fig. 4B; Video S5). Interestingly, cells that are adherent but do not apply protrusive forces did not divide successfully in simulation (Fig. S2). This suggests that the primary advantage of the adherent surface is that it enables cells to apply protrusive forces. Without these, adhesion acts to resist the myosin II forces and prevent sufficient cellular deformation that would otherwise enable cell division to proceed successfully. Defective cytokinesis on adherent surfaces has been documented in several *Dictyostelium* strains that have aberrant actin polymerization. In cells lacking coronin, an actin binding protein, attachment to the surface does not facilitate cell division [24]. Similarly, cells lacking AbiA, a component of the SCAR complex, exhibit deficient cytokinesis in adherent conditions [25].

Beyond the cell's ability to divide in non-adherent conditions, these simulations show some further differences from those of *myoII* null cells. The initial rate of furrow ingression in these simulations is faster than observed in the simulations devoid of myosin II contractile force. This is expected as the initial deformation now includes the cooperative interaction of two force generating subsystems. Differences are also seen in the shape of the daughter cells, as these simulations give rise to rounder cells than cells from simulations that lack myosin II contractile forces. These observations are in agreement with experimentally measured differences between WT and *myoII* null cells (Fig. 4B vs. 3B) [10].

### Strain-stiffening slows down division

Comparing the simulated furrow-thinning trajectory to that measured experimentally in WT cells did reveal some important differences (Figs. 3, 4). The furrows in our simulations exhibit the same sharp drop in radius that is seen in our models of *myoII* null cells, which can be attributed to the large rise in pressure due to the increase in curvature. This sharp drop-off, which is not seen experimentally, leads to faster division than in real cells. To account for this difference we considered the possible role that strain-stiffening may have on furrow ingression. Strain-stiffening is a non-linear effect whereby materials harden when deformed sufficiently; this has been observed in several biopolymers [26]. Hallmarks of strain-stiffening can be seen in other aspects of *Dictyostelium* cellular and cytokinesis mechanics in a myosin II-dependent manner. For example, in response to pressure jumps from micropipette aspiration, cells missing myosin II show non-linear effects that are absent in WT cells, suggesting that myosin II pre-stresses the network, leading to strain-stiffening [12]. We incorporated a phenomenological description of strain-stiffening into our model (Methods) and simulated the system. As expected, the initial rate of furrow ingression was unaffected. However, as the furrow diameter became small enough to cause strain-stiffening, the furrow ingressed more slowly, matching the rates observed experimentally (Fig. 4C–E; Videos S6 and S7). While strain stiffening slows down the cytokinetic progression of WT strains, we have not observed this slowdown in experiments of *myoII* null cells. This suggests that myosin II is a fundamental component that provides this stiffening effect, an observation that is consistent with our measured material properties of *myoII* null cells [8], [12].

Using this full model we considered the effect that changing the material properties of the cell have on the furrow ingression dynamics. For example, we varied the parameter controlling elasticity (*K* in Fig. 1B, according to Equation 11) and simulated furrow ingression (Fig. S3). Increasing the elasticity constant by 40% led to a slower, more linear initial ingression (cross-over time increased from 370 to 420 s), as well as slower division overall (415 to 495 s). In contrast, decreasing the elastic constant 30% shortened the cross-over time (370 to 350 s) as well as the total trajectory (415 to 380 s). The simulated trajectories of the model with reduced elasticity are reminiscent of experiments of cells lacking globally-distributed proteins, such as RacE and dynacortin, that have a strong effect on the viscoelastic moduli and act to slow furrow ingression [10].

Finally, the model allows us to sort out an additional point about cytokinesis furrow ingression dynamics. In particular, it is often thought that *myoII* null cells divide by simply crawling apart. However, our simulations indicate key differences in mitotic cell division for both WT (Fig. 4B,C) and *myoII* null cells (Fig. 3B) and interphase traction-mediated cytofission (Fig. 2B). By plotting the pole-to-pole distance as a function of time (Fig. 4F), it can be seen that interphase cells drive fission solely by crawling apart. This leads to significant pole separation as well as long and thin morphologies (Fig. 2B). In contrast, mitotic cells that have spatial heterogeneity in their mechanical properties initiate division through protrusion, but divide quite differently, with pole-to-pole distances that are similar to WT cells.

## Discussion

Computational modeling presents an opportunity to dissect the different subsystems that contribute to force generation and subsequent cell shape changes during cytokinesis. Using an experimentally validated viscoelastic model of a *Dictyostelium* cell, and relevant measured data on adhesion, protrusion and myosin II-generated contractile forces, we successfully simulated cell division in several distinct virtual strains. We show that cytokinesis can be divided into three distinct phases: 1, initial furrow ingression; 2, Laplace-like pressure dominated, and 3, bridge-dwelling phase [10], [27]. Initial furrow ingression can be achieved in multiple ways using separate subsystems. Adherent cells can pull themselves apart by applying protrusive forces in two opposite directions. Alternatively, in the absence of adhesion, the initial ingression can come from the contractile forces provided by myosin II [28]. We note that alone, both of these subsystems require certain special conditions to complete division; either traction to apply protrusive forces (Fig. 2B) or the absence of resistance from adhesion (Fig. S2). Both our simulations and previous experimental evidence show that *Dictyostelium* cells can initiate cytokinesis using either of these two force producing processes. In other cell types which are less adherent, it is possible that myosin II-driven ingression may play a more important role during this first phase of ingression.

**Figure 5:**
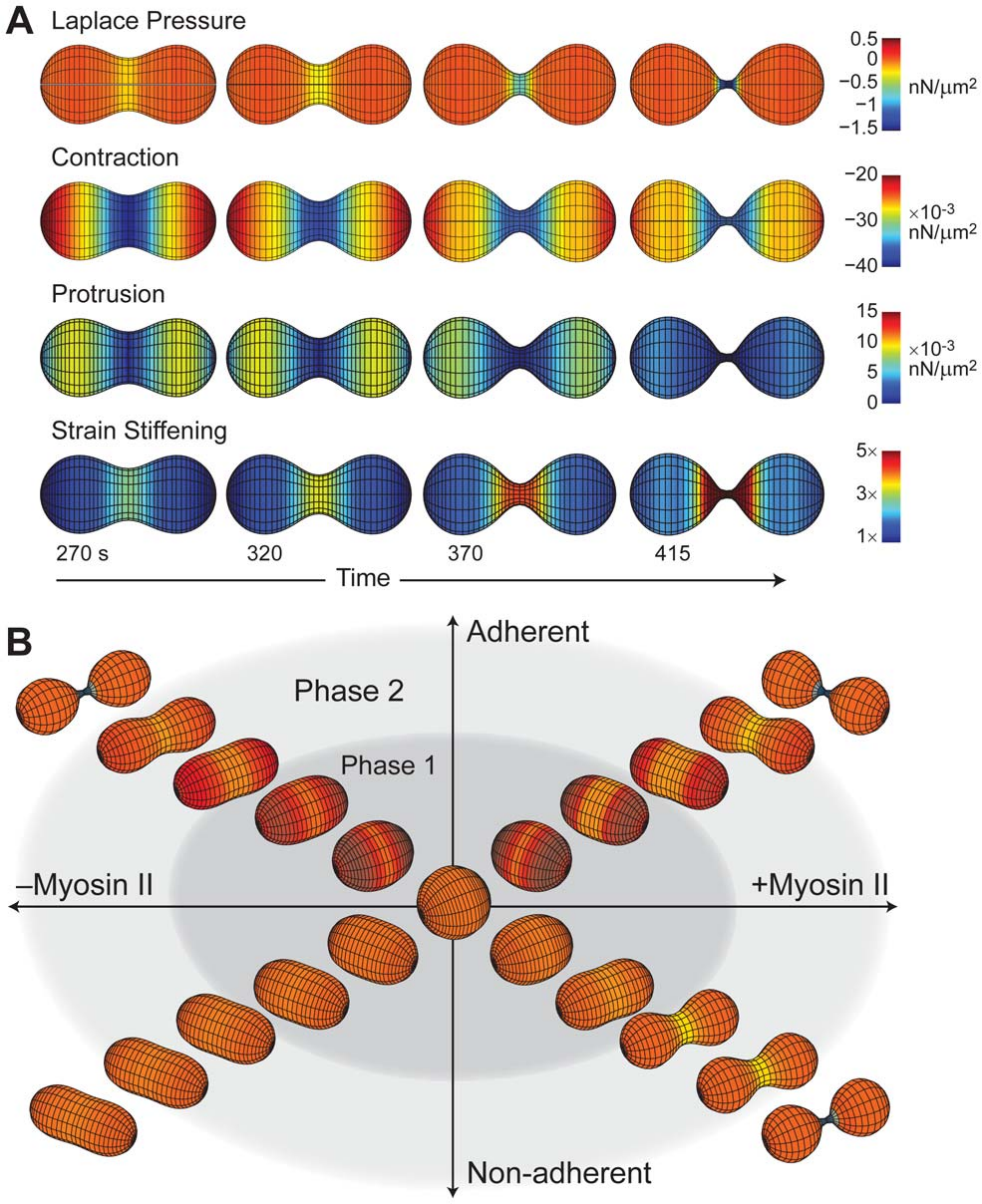
Distribution of stresses acting on the cell. A. Temporal and spatial profiles of different stresses in WT simulation at various time points. Negative stresses denote inward-directed forces. B. Summary of phenotypes observed in the simulations separated by the different conditions applied. Phase 1 denotes the initial breaking of spherical symmetry. Phase 2 is the progression into a dumb-bell shape.

While these subsystems are important to start cytokinesis, the major shape change occurs during phase 2 when the bulk of the force is provided by passive Laplace-like pressure differences that result from induced changes in mean curvature (Fig. 5). Our results demonstrate that either adhesion in combination with protrusive forces or myosin II are sufficient to drive the cell to phase 2 to allow the Laplace-like pressures to take over. Our results are also consistent with experiments of *Dictyostelium* cells flattened by agar overlay where full myosin II mechanochemistry is required to overcome the added mechanical stress from the compression by the sheet of agar [29].

The combination of Laplace pressures and myosin II-generated forces are large enough to make the cell divide faster than what is observed experimentally, suggesting the presence of another component that acts to slow down cell division. Several possibilities exist for this resistive force, including an axial compression acting on the ends of the furrow to counteract the effects of Laplace-like pressures and/or elastic relaxation [10]. More recent observations indicate that the slowdown depends on the lever-arm length of myosin II [30]. Wild type and a longer lever-arm mutant myosin II (2×ELC) lead to furrow-thinning trajectories that are WT-like. In contrast, a short lever-arm mutant deleted for both light chain binding sites (ΔBLCBS) shows *myoII* null-like furrow-thinning trajectory though it accumulates at the cleavage furrow, demonstrating that it is not the presence of myosin II bipolar thick filaments alone that are responsible for the slower WT furrow ingression dynamics. Rather, the lever-arm length dependency suggests that it is the stalling of myosin II in the isometric state that is responsible for the slower ingression dynamics. This locking of the myosin II motor on the actin filaments then leads to an increase in myosin II-mediated crosslinking and tension and consequently an increase in the furrow stiffness (*i.e.* strain-stiffening). While it is difficult to directly quantify the level of this increase or the time-scales over which the strain-stiffening is prominent, our simulations do suggest that non-linear strain-stiffening properties of the cortex may account for the slowdown of furrow ingression. In actuality, all three, compressive stress, elastic relaxation and strain-stiffening, are likely to contribute to varying degrees to the slowdown.

Though most conceptions of cytokinesis contractility have focused almost exclusively on the contractile ring [7], our simulations demonstrate that cell division is the result of multiple force-generating subsystems, acting on the cellular mechanical network. This explanation is particularly compelling because our model, using only experimentally measured parameters, accurately reproduces WT and mutant cell division events.

While it is often considered that cytokinesis is regulated spatiotemporally by linear biochemical pathways (such as by small GTPases and kinases), another level of control is equally important. For example, myosin II not only generates contractility but also controls the cortical tension, elastic modulus, and strain-stiffening. Thus, myosin II regulation affects both a force-generating subsystem and the mechanical network on which the force acts, highlighting the complex nature of the system.

## Methods

To simulate furrow ingression we account for the forces that are active during mitosis as well as a physical model of the cell. We also need a modeling framework capable of simulating cellular deformations. Previously, we demonstrated that cell shape changes can be recreated accurately using the level set formalism, coupled with a viscoelastic model of a cell and a description of forces acting on the cell [14]. Table 2 presents the nominal model parameters, and Table 3 presents a summary of the algorithm used.

**Table 2:** Nominal Parameters.

Parameter	Value	Unit	Reference
Time step	5	ms	
Grid size	0.1	µm	
Nominal cortical elasticity (*K*)	0.098	nN/µm^3^	[14]
Cortical viscosity (*D*)	0.064	nN-s/µm^3^	[14]
Cytoplasm viscosity (*B*)	6.1	nN-s/µm^3^	[14]
Maximum adhesion (σ*_adh-max_*)	0.05	nN/µm^2^	[32]
Maximum protrusive stress (σ*_pro-max_*)	0.71	nN/µm^2^	[42], [43]
Maximum contractile stress (σ*_myo-max_*)	0.04	nN/µm^2^	[10]
Surface tension at pole (γ_pole_)	1	nN/µm	[12]
Surface tension at furrow (γ_furrow_)	1–1.8	nN/µm	[12]
Initial cell radius (*R*_0_)	5.0	µm	[10]
Volume regulation constant (*K_vol_*)	0.1	nN/µm^5^	[14]

**Table 3:** Algorithm steps.

Initialization	
Initialize the LSM potential function	φ(***x***,0) = signd(***x***,Γ), ***x*** **∈R** ^2^
Initialize the cell's viscoelastic state	*l*(***x***,0) = 0; *x_m_*(***x***,0) = 0, where ***x*** **∈**Γ

### Level set method

The level set method takes an Eulerian approach, tracking a moving boundary (denoted Γ(*t*)) on a static Cartesian grid deformed by a continuum stress field across the simulation domain [13]. In our simulations, we take a two-dimensional domain and assume cylindrical symmetry about the division axis (Fig. 1A). The level set formalism defines a potential function φ(***x***,*t*) for which the boundary is the zero-level set: Γ(*t*) = {***x*** **∈R** ^2^ | φ(***x***,*t*) = 0}. In our simulations, we initialize the potential function with the signed distance function, whose magnitude equals the shortest distance from a point ***x*** **∈R** ^2^ to the curve Γ(*t*) and whose sign is positive if the point is outside the cell and negative otherwise. In practice, as the potential function evolves over time, it can become quite steep or flat, leading to numerical errors. These can be minimized by re-initializing the potential function periodically using the equation
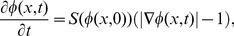
(1)where *S*(*φ*(*x*,0)) is taken as +1 inside the cell, −1 outside the cell and zero on the cell membrane.

The potential function evolves according to the Hamilton-Jacobi equation
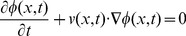
(2)The vector *v*(*x*,*t*) is the velocity of the level set moving in the outward normal direction which, in our simulations, describes the cell's membrane protrusion and retraction velocities. These are driven by a combination of active and passive stresses acting on a mechanical model of the cell, to be described next.

### Mechanical model

Previously we developed a mechanical description of a cell in the level set framework and fitted a viscoelastic model topology with parameters obtained from measurements of cells deformed using micropipette aspiration [14]. The model assumes that the cell deformation obeys 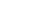, where ***v*** is the velocity defined above, and *x_m_* is the displacement of the membrane (Fig. 1A, B). The total membrane displacement is the sum of the displacements of the cortex (*x_cor_*) and cytoplasm (*x_cyt_*). To describe how stresses affect these, we use a Voigt model, which consists of the parallel connection of elastic (*K*) and viscous (*D*) elements, to represent the cortex connecting the cell membrane and the cytoplasm (Fig. 1B). The viscous component describes the association and dissociation dynamics of actin cross-linkers. The cytoplasm is modeled by a purely viscous element (*B*) placed in series with the Voigt element. In our simulations, we use stress rather than force to drive the cellular deformations thus accounting for the extra µm^2^ found in the parameters in our model. The model assumes that these displacements occur normal to the cell surface. This neglects bending effects, which are relevant at much smaller length-scales than those we consider in modeling cytokinesis [27], [31].

In the simulations, the total stress (σ*_tot_*) is applied at the cell boundary, according to: 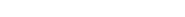, where *x_cor_* and *x_cyt_* represent the positions of the cortex and cytoplasm, respectively. Using the membrane displacement, *x_m_* = *x_cor_*+*x_cyt_*, we can rewrite the system of equations as
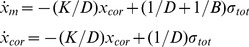
(3)We thus obtain the membrane velocity solving first for *x_cor_* and then for 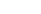. This value is entered into the Hamilton-Jacobi Equation (Eqn. 2).

### Stresses acting on the cell

The total net stress (σ*_tot_*) is computed for the simulation domain as the vector sum of all stresses acting on the cell. This includes stress contributions from active components, adhesion (σ*_adh_*), protrusion (σ*_pro_*), and myosin-based contraction (σ*_myo_*), as well as passive components due to surface tension (σ*_ten_*) and volume regulation (σ*_vol_*). Thus
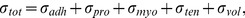
(4)These individual components are now described in detail.

### Adhesion model

Our model of adhesion uses a continuum stress field to counteract cellular deformations [32] and is based on defining an adhesion map, as previously described [33]. Though our simulations assume that the cell has cylindrical symmetry, for the purposes of computing adhesion and protrusion, we instead consider the cross-sectional area in the (*z*,*r*) plane, which more closely corresponds to the contact area between cell and substrate. We compute area densities in both *r* and *z* directions, normalized to the total cell cross-sectional area:
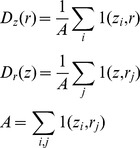
(5)Here **1**(*z*,*r*) is the indicator function that equals one when the point (*z*,*r*) is inside the cell and zero otherwise, and the summations are done over all simulation points in either the *z*- or *r*-direction (Fig. 1C). These densities describe the fraction of the cell-substrate contact area that lie in the respective strips either in the *z*- or *r*-directions. We multiply these two densities and scale by the maximum adhesion stress (σ*_adh-max_*) to generate a spatial adhesion map:
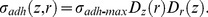
(6)This adhesion is applied spatially as a resistive stress element that counteracts the net effect of the other stresses. To evaluate this new model we simulated the cellular response to a series of pulses (Fig. S4) and compared this response to that of the nominal model. Simulations that incorporate adhesion show a delayed initial response and these cells also take longer to reach steady state.

### Protrusion model

We incorporate protrusive forces based on several assumptions (Fig. 1D). First, protrusion acts at both ends of the cell to drive the cell apart. Thus, the protrusive stress acts away from the *z* = 0 line. Second, the local protrusive forces depend on the contact area (as calculated by *D_r_*(*z*) above) and increase as you move away from the cleavage furrow (scaled by a linear function *l*(*z*) with values of zero at the center of the division axis (*z* = 0) and one at the poles). Finally, the protrusive force decreases over time as the cell is dividing. We incorporate this by including an exponential function indexed by the furrow diameter (*w_f_*(*t*), defined as the diameter of the cell at the midpoint along the *z*-axis). Together, these assumptions lead to a protrusion stress whose magnitude is given by
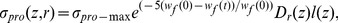
(7)where σ*_pro-max_* is maximum stress applied (Table 2). Though the stress is assumed to act along the *z*-axis (Fig. 1D), only the component normal to the surface is used in the simulations. The model used here is phenomenological, but captures the net movement of the membrane away from the division plane. Other approaches, which look at finer scale effects for modeling protrusion, have been considered in the literature [34], [35].

### Myosin II contractile force

An active contractile force from the work of myosin II against the cytoskeleton is present in wild type cells. This force acts tangentially to the cortex, thereby constricting the cell and, because we assume cylindrical symmetry, this reduces the circumference (Fig. 1E). This has the net effect of reducing the furrow diameter (with a stress reduced by a factor of 2π to account for conversion from circumference to radius). Thus, to incorporate this into our model, we assume that the contractile stress acts radially inward. The magnitude of the local force depends on two things, the maximum stress generated by myosin II and the local distribution of myosin II.

To compute the maximum stress we note that if we assume 3.4 µM total cellular concentration of myosin II monomers (each monomer is composed of two heavy chains, two essential light chains and two regulatory light chains) [11], [36], then a mitotic cell with a radius of 5 µm contains 1×10^6^ myosin monomers (2×10^6^ heads). Given the *Dictyostelium* myosin II unloaded duty ratio (0.6%) and the force generated by the power stroke of the myosin (3 pN), the maximum total force that can be generated from myosin II is ∼40 nN, assuming no load-dependent shifts in the duty ratio. Because only ∼20% of the myosin II is found in the assembled bipolar thick filament state, most of which resides in the cortex [11], [37], the resulting maximal force is 10 nN. This number is used to compute the total maximum stress by dividing by the cellular area (4π*R* ^2^).

To apportion this stress spatially, we imaged *myoII::*GFP-myoII cells (*mhcA* (HS1):: pBIG:GFP-myosin II; pDRH:RFP-tubulin) undergoing cytokinesis as previously described [38]. From this movie, the GFP-myosin II fluorescent intensities were extracted to quantify myosin density. Cell images were aligned by their centroids and along the division axis. For each image, edge detection was performed to identify cell periphery. Using this edge, the GFP-myosin II intensity was computed for 5 pixels (1 µm) inwardly normal from the boundary, a region likely to contain cortical myosin. An average of these intensities was assigned as the local myosin density at that boundary point. The cell shape was averaged across both its axes of symmetry along with the GFP-myosin II distributions to construct a symmetric myosin profile along the division axis. This profile was smoothed using a cubic smoothing spline. For each image in the time series, a one-dimensional profile was constructed, indexed to the position along the division axis and the measured furrow diameter (Fig. S1). The resultant map (*myo*(*r*,*z*)) describes the distribution of myosin as a function of radius and is used to generate a stress:
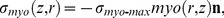
(8)where **n** is the outward normal unit vector.

### Surface tension

Local differences in mean curvature and surface tension give rise to spatially heterogeneous stresses on the cell. The stress differential across the boundary, described by the Young-Laplace relationship, is given by σ*_ten_* = γ(*z*)*κ_mean_*(*z*)**n**, where γ(*z*) describes the local cortical tension, *κ_mean_* is the mean curvature and ***n*** is a normal unit vector.

The mean curvature, *κ_mean_*, is the arithmetic mean of two principal curvatures (*κ_mean_* = ½(*κ*_2*D*_+*κ_P_*)) [39]. The first is computed using a Lagrangian formulation based on the cellular boundary: *κ*_2*D*_(*x*, *y*) = (*x*′*y*″−*y*′ *x*″)/(*x*′^2^+*y*′^2^)^3/2^ where the point (*x*,*y*)**∈**Γ. The primes denote spatial derivatives along the boundary and are approximated by the center weighted difference between two points [13]. The computation of the second principal curvature takes advantage of the cell's cylindrical symmetry: *κ_P_* = *N_r_*(*r*)/*r*, where *N_r_*(*r*) is the normal in the radial direction at a given point, and *r* is the radius of the cell at that location [39].

For interphase cells, we assume that cortical tension is homogeneous around the cell with a nominal value of 1 nN/µm [10]. For mitotic *myoII* null cells, we assume a spatially heterogeneous γ with values of 0.5 and 1.0 nN/µm at the pole and furrow, respectively [8], [12]. We interpolate these values using a Gaussian profile:
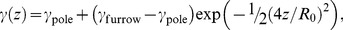
(9)where *R*_0_ is the initial radius of the cell and *z* is the horizontal position between the pole and furrow. In wild type cells, the cortical tension at the pole and furrow are 1 and 1.8 nN/µm, respectively [10]. In these simulations, we interpolate between these two values according to the measured myosin II concentration (described below). This profile is used as a means of marking intracellular changes in the material properties of the cell during division, not necessarily implying that surface tension comes from myosin. We considered other schemes for spatially varying the cortical tension, but all gave similar results. For example, simulations of cells lacking myosin contractility were run varying cortical tension using a Gaussian distribution. Additionally, we performed simulations using both the myosin density profile and a normal distribution to simulate the surface tension profile but found little difference between the two.

### Volume conservation

We assume that the cellular volume remains constant [14]. To enforce this constraint we implement a stress
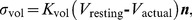
(10)where *n* is the outward normal. The cell's volume is evaluated by assuming the cell is radially symmetric: *V*_actual_ = ∫_cell length_π*r*(*z*) *dz*. Large values of *K*_vol_ keep the cell volume relatively constant, but can lead to small oscillations as the stress overshoots the required target. In our simulations, we set *K*_vol_ = 0.1 nN/µm^5^, which was sufficiently high to ensure that both volume changes were small but maintained the stability of the simulations, though some oscillations (as seen in the furrow measurements in Fig. 3E) do appear.

### Strain stiffening

We assume that the elastic component of the cell undergoes strain stiffening. Though no precise model for strain stiffening is currently available, in *Dictyostelium* cells, we have previously observed the effect of nonlinearities in cellular responses to deformations of varying size. These differences depend on the presence of myosin II, likely due to stalling of the myosin II motors [12], [30]. Hence, we posit a plausible phenomenological model of strain stiffening that includes the effect of both the strain (by incorporating the change in the furrow diameter) and the local myosin II-density. The increased elasticity at point *x* is given by
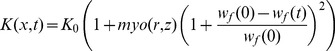
(11)where *K*_0_ is the nominal elasticity (Table 3), *myo*(*z*,*r*) is the myosin density profile (described above) and *w_f_*(*t*) is the furrow diameter. The resulting temporally and spatially varying map of elasticity is then applied to the material model.

### Implementation

The simulations are based on the Level Set Toolbox [40] and are coded in Matlab (Mathworks, Natick, MA). The code is extended to implement the local level set algorithm [41], a modification of the level set method that decreases the computational complexity by solving quantities only near the boundary. Simulations were implemented on a dynamic grid of fixed height (12 µm) and varying width (12–24 µm), with density of 20 points/µm and 5-ms time steps. Simulation takes approximately 2 hours for every minute of cell division on a desk top PC.

### Furrow-thinning dynamics

Strains used to determine experimental furrow thinning trajectories are the *myoII* null (*mhcA* (HS1):: pLD1A15SN; pDRH:GFP-tubulin) and the rescued *myoII* null as WT (*mhcA* (HS1):: pBIG:GFP-myosin II; pDRH:GFP-tubulin) [8], [12]. Time-lapse DIC images of were taken at 2-s intervals with a 40× (N.A. 1.3) objective with 1.6× optivar [8], [12]. To determine the relative furrow diameter, we find the furrow diameter (*w_f_*(*t*), the diameter of the cell at the midpoint along the *z-*axis) and the furrow length (*L_f_*(*t*), the distance between the points of inflection in the furrow region). The point when the two are equal is the cross-over time, *t*_x_ and this marks the cross-over distance (*D*_x_ = *w_f_*(*t*_x_) = *L_f_*(*t*_x_)). We define the relative furrow diameter as the ratio *w_f_*(*t*_x_)*/D*_x_. Rescaled time is defined by shifting time so that *t*_x_ = 0. Furrow diameter and length at each time point were measured using ImageJ (http://rsbweb.nih.gov/ij/).

## Supporting Information

Figure S1 **Distribution of GFP-myosin II motors during cytokinesis.** A. Dividing cells were imaged at five time points approximately 100 seconds apart during cytokinesis, and the fluorescence intensity was measured around the cell perimeter. B. Furrow diameter as a function of time. During the simulation, the furrow diameter is measured to determine where the cell is along this profile. C. Spatial distribution of myosin II motors along the division axis (*z*) at different time points. During the simulation, the myosin II forces were distributed according to these profiles indexed by the furrow diameter.
(PDF)

Figure S2 **Simulation of furrow ingression with no protrusion.** A. This cell model includes myosin II contractile forces, adhesion, but no protrusive forces. As shown, these cells stalled. B. Comparison of furrow diameter between simulations in panel A with WT dynamics (reproduced from Fig. 3B).
(PDF)

Figure S3 **Furrow thinning trajectory for varying elasticities.** Elastic constant (*K* in Fig. 5B) was increased (+40%) and decreased (−30%) and the resultant furrow thinning dynamics were compared to the nominal (WT) model.
(PDF)

Figure S4 **Effect of adhesion.** System response to step applications (σ_step_) of 1 nN/µm^2^, for various levels of adhesion (ranging from 0 to 100% of maximum). Simulations that incorporate greater adhesion show a delayed initial response to the stress. These cells also take longer to reach steady state after removal of the stress.
(PDF)

Figure S5 **Profiles of different stresses at various time points for an adherent** ***myoII*** **null cell.** Negative stresses denote inward-directed forces.
(PDF)

Video S1 Simulation of model incorporating adhesion and protrusion forces.
(AVI)

Video S2 Simulation of model incorporating spatially heterogeneous cortical tension, adhesion and protrusion.
(AVI)

Video S3 A *myoII* cell undergoing cytokinesis. Movies were collected with two second intervals.
(AVI)

Video S4 Simulation of model incorporating spatially heterogeneous cortical tension and myosin II-dependent contractile force.
(AVI)

Video S5 Simulation of model incorporating spatially heterogeneous cortical tension, myosin II-dependent contractile force, adhesion, and protrusion.
(AVI)

Video S6 Simulation of model incorporating spatially heterogeneous cortical tension, myosin II-dependent contractile force, adhesion, protrusion, and strain stiffening.
(AVI)

Video S7 A WT cell undergoing cytokinesis. Movies were collected with two second intervals.
(AVI)
