# Supplementary figures and images for: Deconvolution of the Cellular Force-Generating Subsystems that Govern Cytokinesis Furrow Ingression
Source: PLoS Comput Biol. 2012 Apr 26;8(4):e1002467. doi: 10.1371/journal.pcbi.1002467 (PMC3343096; doi:10.1371/journal.pcbi.1002467)

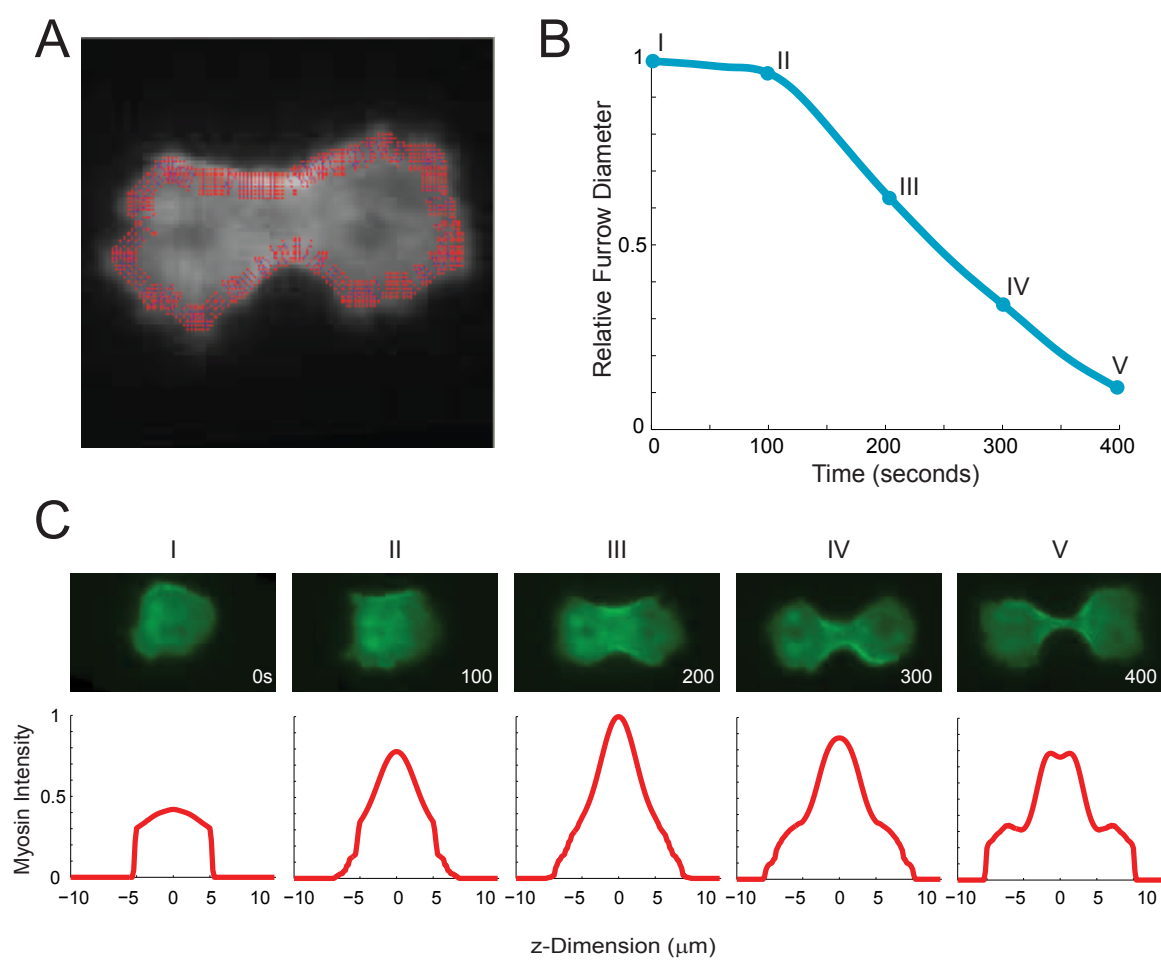

Poirier et al.  
Supporting Figure S1

Supplement: Figure S1 — Distribution of GFP-myosin II motors during cytokinesis. A. Dividing cells were imaged at five time points approximately 100 seconds apart during cytokinesis, and the fluorescence intensity was measured around the cell perimeter. B. Furrow diameter as a function of time. During the simulation, the furrow diameter is measured to determine where the cell is along this profile. C. Spatial distribution of myosin II motors along the division axis (z) at different time points. During the simulation, the myosin II forces were distributed according to these profiles indexed by the furrow diameter. (PDF) [file pcbi.1002467.s001.pdf]

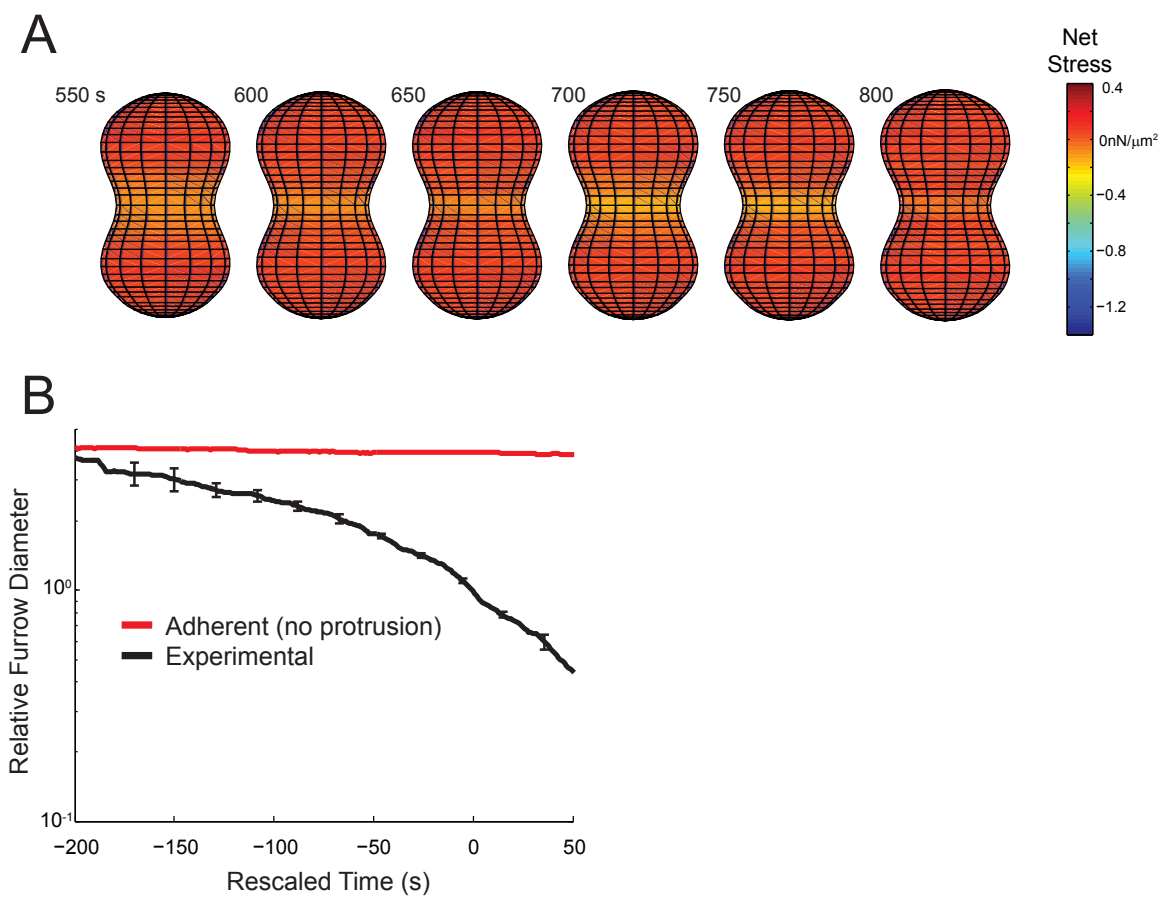

Poirier et al.  
Supporting Figure S2

Supplement: Figure S2 — Simulation of furrow ingression with no protrusion. A. This cell model includes myosin II contractile forces, adhesion, but no protrusive forces. As shown, these cells stalled. B. Comparison of furrow diameter between simulations in panel A with WT dynamics (reproduced from Fig. 3B). (PDF) [file pcbi.1002467.s002.pdf]

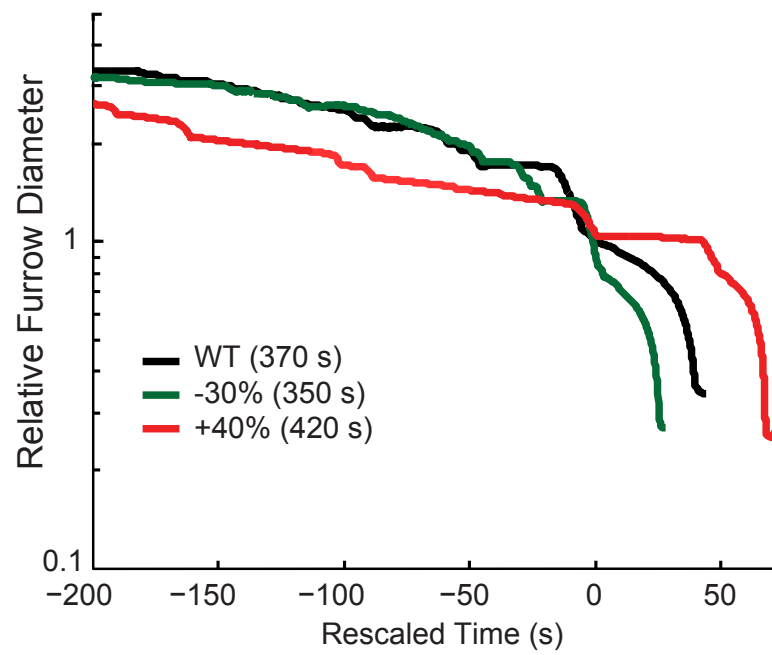

Poirier et al.  
Supporting Figure S3

Supplement: Figure S3 — Furrow thinning trajectory for varying elasticities. Elastic constant (K in Fig. 5B) was increased (+40%) and decreased (−30%) and the resultant furrow thinning dynamics were compared to the nominal (WT) model. (PDF) [file pcbi.1002467.s003.pdf]

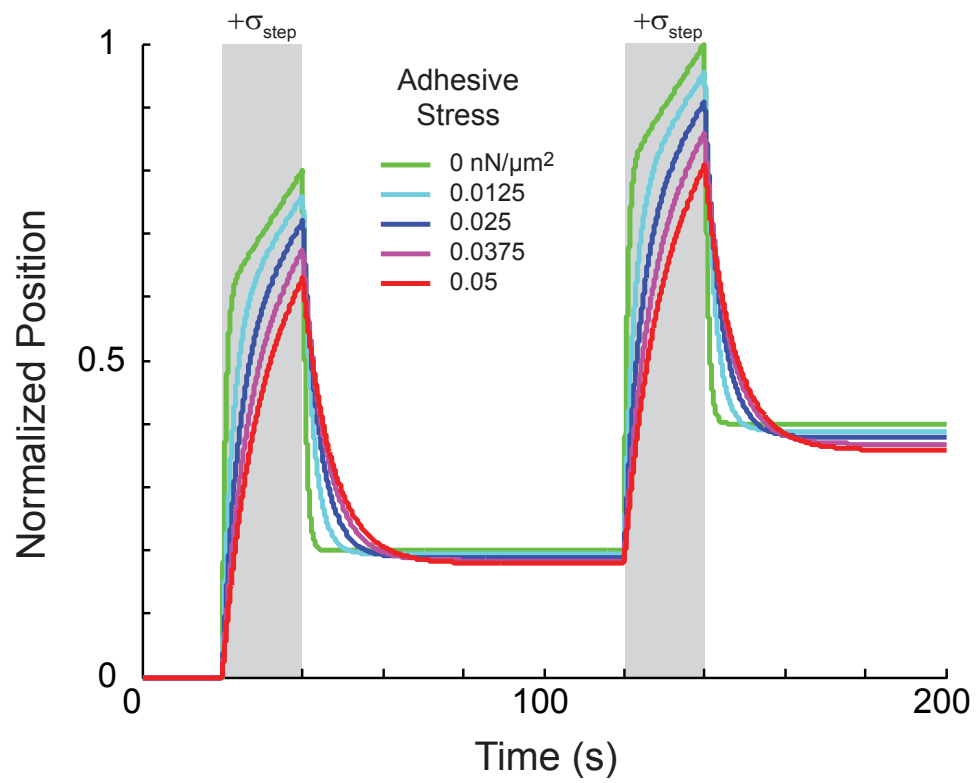

Poirier et al.  
Supporting Figure S4

Supplement: Figure S4 — Effect of adhesion. System response to step applications (σstep) of 1 nN/µm2, for various levels of adhesion (ranging from 0 to 100% of maximum). Simulations that incorporate greater adhesion show a delayed initial response to the stress. These cells also take longer to reach steady state after removal of the stress. (PDF) [file pcbi.1002467.s004.pdf]

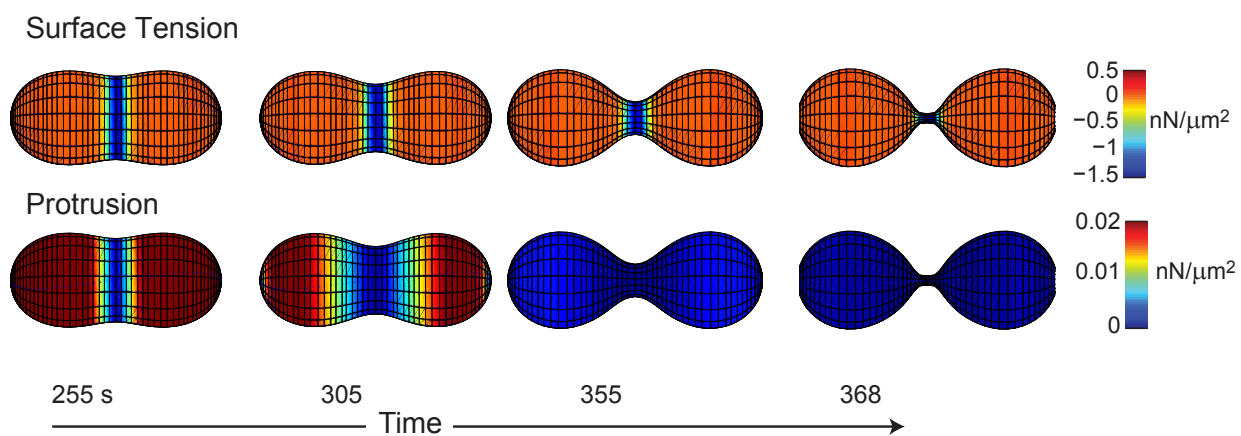

Poirier et al.  
Supporting Figure S5

Supplement: Figure S5 — Profiles of different stresses at various time points for an adherent myoII null cell. Negative stresses denote inward-directed forces. (PDF) [file pcbi.1002467.s005.pdf]
